# GnRH antagonist treatment of malignant adrenocortical tumors

**DOI:** 10.1530/ERC-17-0399

**Published:** 2018-08-21

**Authors:** Milena Doroszko, Marcin Chrusciel, Joanna Stelmaszewska, Tomasz Slezak, Slawomir Anisimowicz, Ursula Plöckinger, Marcus Quinkler, Marco Bonomi, Slawomir Wolczynski, Ilpo Huhtaniemi, Jorma Toppari, Nafis A Rahman

**Affiliations:** 1 Institute of BiomedicineUniversity of Turku, Turku, Finland; 2 Department of Reproduction and Gynecological EndocrinologyMedical University of Bialystok, Bialystok, Poland; 3 Department of Biochemistry and Molecular BiologyUniversity of Chicago, Chicago, Illinois, USA; 4 Center of Gynecology and Reproductive Endocrinology ArtemidaBialystok, Poland; 5 Interdisciplinary Center of Metabolism: EndocrinologyDiabetes and Metabolism, Charité University Medicine Berlin, Berlin, Germany; 6 Endocrinology in CharlottenburgBerlin, Germany; 7 Department of Clinical EndocrinologyCharité Campus Mitte, Charité University Medicine Berlin, Berlin, Germany; 8 Department of Clinical Sciences & Community HealthUniversity of Milan, Milan, Italy; 9 Department of Surgery and CancerFaculty of Medicine, Imperial College London, London, U.K.; 10 Department of PediatricsTurku University Hospital, Turku, Finland

**Keywords:** GNRHR, LHCGR, GnRH antagonist, therapy, cetrorelix

## Abstract

Aberrantly expressed G protein-coupled receptors in tumors are considered as potential therapeutic targets. We analyzed the expressions of receptors of gonadotropin-releasing hormone (GNRHR), luteinizing hormone/chorionic gonadotropin (LHCGR) and follicle-stimulating hormone (FSHR) in human adrenocortical carcinomas and assessed their response to GnRH antagonist therapy. We further studied the effects of the GnRH antagonist cetrorelix acetate (CTX) on cultured adrenocortical tumor (ACT) cells (mouse Cα1 and Y-1, and human H295R), and *in vivo* in transgenic mice (SV40 T-antigen expression under inhibin α promoter) bearing *Lhcgr* and *Gnrhr* in ACT. Both models were treated with control (CT), CTX, human chorionic gonadotropin (hCG) or CTX+hCG, and their growth and transcriptional changes were analyzed. *In situ* hybridization and qPCR analysis of human adrenocortical carcinomas (*n* = 11–13) showed expression of *GNRHR* in 54/73%, *LHCGR* in 77/100% and *FSHR* in 0%, respectively. CTX treatment *in vitro* decreased cell viability and proliferation, and increased caspase 3/7 activity in all treated cells. *In vivo*, CTX and CTX+hCG (but not hCG alone) decreased ACT weights and serum LH and progesterone concentrations. CTX treatment downregulated the tumor markers *Lhcgr* and *Gata4*. Upregulated genes included *Grb10*, *Rerg*, *Nfatc* and *Gnas*, all recently found to be abundantly expressed in healthy adrenal vs ACT. Our data suggest that CTX treatment may improve the therapy of human adrenocortical carcinomas by direct action on GNRHR-positive cancer cells inducing apoptosis and/or reducing gonadotropin release, directing tumor cells towards a healthy adrenal gene expression profile.

## Introduction

Adrenocortical adenomas can be detected in around 5% of the population over the age of 50 (Thompson & Young Jr 2003), whereas fatal adrenocortical carcinomas are yearly diagnosed in 1–2 patients per million (Allolio & Fassnacht 2006). Adrenocortical carcinomas have a bimodal distribution, clustering in children under 10 years and adults aged 40–50 years, being 1.5-fold more common in women than men (Allolio & Fassnacht 2006). Treatment of these cancers mainly involves surgery, adjuvant with mitotane and/or chemotherapy, postoperative radiation and treatment of the hormone excess (Creemers* et al.* 2016). These forms of therapy remain inefficient, resulting in 5-year survival of only 10–25% (Allolio & Fassnacht 2006, Fassnacht & Allolio 2009).

Ectopic expression of reproductive hormone G-protein coupled receptors (GPCR), such as luteinizing hormone/chorionic gonadotropin (LHCGR) or gonadotropin-releasing hormone (GNRHR), has been reported in malignancies of reproductive organs (i.e. ovarian, breast or prostate) (Huhtaniemi 2010, Ghanghoria* et al.* 2016) as well as in adrenocortical disorders, such as adrenocorticortopin-independent adrenal hyperplasia, aldosterone-producing ACA and pregnancy-induced Cushing syndrome (Carlson 2007, Ziegler* et al.* 2009, Huhtaniemi 2010, Albiger* et al.* 2011, Plöckinger* et al.* 2017). Therefore, treatment with GnRH analogues, to block gonadotropin secretion, could provide a therapeutic strategy for the above-mentioned tumors (Limonta* et al.* 2012, Ghanghoria* et al.* 2016). GnRH analogues have also been shown to act directly on GNRHR expressing cells and to promote (splenocytes, thymocytes and lymphocytes) or inhibit the growth of normal (ovarian granulosa cells) (Park* et al.* 2013) and tumorous (prostate, breast, ovary, endometrium, adrenal, lung, pancreatic, melanoma, glioblastoma) cells (Ziegler* et al.* 2009, Jääskeläinen* et al.* 2010, Limonta* et al.* 2012, Park* et al.* 2013, Seitz* et al.* 2014). Interestingly, even though the signaling mechanisms of GnRH agonists and antagonists in pituitary cells differ, their direct actions on tumor cells may be similar (Limonta* et al.* 2012, Ghanghoria* et al.* 2016). Main effects of GnRH analogue treatment on tumor cells are the inhibition of proliferation, metastatic potential and angiogenesis (Limonta* et al.* 2012, Ghanghoria* et al.* 2016). Previous *in vitro* and *in vivo* xenograft studies have had important pitfalls. Firstly, these models did not recapitulate the complicated structure of tumor tissue, and, secondly, they lacked the fully functional immune system. Therefore, more accurate assessment of the anti-tumoral efficacy of GnRH analogues *in vivo* necessitates the inclusion of animals naturally developing tumors and with intact immune system.

Inhα/Tag mice, expressing Simian Virus 40T antigen under the inhibin α promoter, and with an intact immune system, are an example of a mouse model developing tumors (Kananen* et al.* 1995, 1996, Chrusciel* et al.* 2014). Intact inhα/Tag mice develop gonadal tumors, but when prepubertally gonadectomized, adrenocortical tumors appear with a hyperplasia-adenoma-adenocarcinoma sequence and abundant LHCGR expression (Kananen* et al.* 1995, 1996, Rilianawati* et al.* 1998, Rahman* et al.* 2001, 2004, Bodek* et al.* 2005, Vuorenoja* et al.* 2007, 2008, 2009, Chrusciel* et al.* 2014, Doroszko* et al.* 2017*a*,*b*). The formation of gonadal and adrenocortical tumors in these mice is gonadotropin dependent, as gonadotropin genetic and pharmacological ablation prevented tumor growth (Rilianawati* et al.* 1999). Furthermore, elevated LH levels through cross-breeding to LHβ subunit overexpressing mice (LHβCT mice) (Risma* et al.* 1995) resulted in simultaneous occurrence of gonadal and adrenocortical tumors (Mikola* et al.* 2003). Our recent findings on inhα/Tag mice showed that, besides LHCGR, the adrenocortical tumors express *Gnrhr* (Doroszko* et al.* 2017*a*), and SV40Tag expression alone is only able to cause adrenocortical hyperplasia but not adenomas (Doroszko *et al.* 2017*b*). The transition from hyperplasia to adenoma required LH/LHCGR signaling, but thereafter tumor progression became gonadotropin independent (Doroszko *et al.* 2017*b*).

Hereby, we revisited the expression of several GPCRs in human adrenocortical carcinomas and mouse adrenal tumors and analyzed further the molecular mechanisms of the GnRH antagonist action on adrenocortical tumor cells *in vitro* and *in vivo*.

## Materials and methods

### Tissue samples

Formalin-fixed paraffin blocks from human adrenocortical carcinomas (*n* = 13) were obtained from the archive of the Department of Pathology, Charité Berlin, Germany; the clinical information such as age, sex and ENSAT score information from the Department of Clinical Endocrinology, Charité Berlin, Germany. The study has been approved by the Ethics Committee of the Charité University Hospital, Germany (No. EA1/169/08), and all patients had provided written informed consent. Formalin-fixed paraffin-embedded tissues were sectioned 5 ± 1 μm and stored in the darkness at +4°C for future staining.

### Immunohistochemistry

Localization of proteins was assessed by immunohistochemistry. Antigens were retrieved using 2100 Antigen Retriever (Aptum Biologics Ltd., Southampton, UK) in Tris–EDTA buffer (pH 9, MKI67) or citrate buffer (pH6). Samples were washed in TBS with 0.1% Tween20 (TBST) (#P1379, Sigma-Aldrich) and blocked for 1 h in 3% BSA in TBST at room temperature (RT). Antibodies against MKI67 (#MIB-1, Dako, diluted 1:500), GNRHR (#19950-1-AP, Proteintech, diluted 1:1000), LHCGR (clone 4G2, antibody donated by Dr Marco Bonomi); supernatant diluted 1:8 (Bonomi* et al.* 2006) or FSHR323 (donated by Dr N. Ghinea) (Vannier* et al.* 1996) at the concentration of 0.5 µg/mL, were applied on the slides and incubated overnight in 4°C. Endogenous peroxidase activity was quenched by 10-min incubation in 3% hydrogen peroxide (Sigma-Aldrich). Depending on the primary antibody host, Dako EnVision+ System-HRP polymer anti-mouse (K4007, Dako) or anti-rabbit (K4011, Dako) were applied, and visualized with Liquid DAB + Substrate Chromogen System (Dako). Slides were scanned by Pannoramic 250 Slide Scanner (3DHISTECH Ltd., Budapest, Hungary) and images were taken using Pannoramic Viewer (3DHISTECH Ltd.). The percentage of MKI67-stained cells was assessed using ImmunoRatio web application (http://153.1.200.58:8080/immunoratio/) (Tuominen* et al.* 2010) from four representative images of each sample.

### *In situ* hybridization

*In situ* hybridization (ISH) was performed using RNAscope 2.5 HD Reagent Kit-BROWN (Advanced Cell Diagnostics, Newark, CA, USA) (Wang* et al.* 2012) with predesigned probes for *GNRHR* (#407999), *LHCGR* (#300031), *FSHR* (#408101), positive control *POLR2A* (#310451) and nonsense dapB (from *Bacillus* S., #310043). Hybridization was performed according to manufacturer’s protocol in HybEZ Oven (Advanced Cell Diagnostics). Slides were scanned by Pannoramic Midi FL slide scanner (3DHISTECH Ltd.) and pictures were taken using Pannoramic Viewer (3DHISTECH Ltd.).

### In vitro

#### Cell culture

Cα1 (Kananen* et al.* 1996) cell line was established from a founder female adrenocortical tumor of C57Bl/6 strain genetic background mouse. Y-1 (ATCC) was derived from a minimally deviated tumor that arose in an adult LAF1 (C57L × A/HeJ) male mouse, following an exposure of the mouse to the radiation of an atomic blast (Cohen* et al.* 1957). Human H295R (ATCC) cell line was isolated from a female adrenocortical carcinoma patient (Rainey* et al.* 1994). These cell lines used in our study were mycoplasma-free. DMEM/F12 (#D2906, Sigma-Aldrich) culture media containing 5 U/mL of penicilin/streptomycin (#15140-122, Fisher Scientific) were supplemented for each cell line as follows, Cα1 10% fetal bovine serum (FBS); Y-1 15% fetal horse serum (FHS) and 2.5% FCS; H295R 2.5% NuSerum (#355100, Corning, New York, NY, USA) and 1× Corning ITS Premix Universal Culture Supplement (#354352, Corning).

Cetrorelix acetate (#C5249, Sigma-Aldrich) was dissolved in 0.1% DMSO (#D8418, Sigma-Aldrich). Recombinant hCG was kindly donated by Organon (Oss, Netherlands). Concentrations of cetrorelix acetate and hCG were validated and set for 10 µM and 10 ng/mL respectively. For all experiments, Y-1 and H295R cells were seeded and stimulated in full culture media, whereas Cα1 cells in medium containing 2.5% FBS. After attaching overnight medium was changed to the medium containing 0.1% DMSO as control (CT), 10 µM CTX (CTX), 10 µM CTX and 10 ng/mL of hCG (CTX + hCG) or 10 ng/mL of hCG (hCG) solutions.

#### Viability test

Viability of the cells was checked using MTS CellTiter 96 Aqueous Non-Radioactive Cell Proliferation Assay (Promega). In brief, cells were seeded on 24-well plates (Cα1 = 120,000 cells/well, Y-1 = 180,000 cells/well, H295R = 180,000 cells/well) and after 48 h of treatment medium was changed for DMEM/F12 medium containing MTS reagent. Cells were incubated for 240 min in 37°C and absorbance at 495 nm was read using Wallac 1420 Victor2 Microplate Reader (Perkin Elmer).

#### Proliferation test

Cells were seeded on 96-well plate (Cα1 = 4000 cells/well, Y-1 = 8000 cells/well, H295R = 8000 cells/well) and after 48 h of treatment medium was decanted and plates frozen in −80°C overnight. After thawing, CyQUANT Cell Proliferation Assay Kit (#C7026, Life Technologies) was applied and DNA standard curves for each cell line were prepared according to manufacturer’s instructions. Fluorescence was read at Ex/Em = 480 nm/520 nm with Wallac 1420 Victor2 Microplate Reader (Perkin Elmer). Results were recalculated using determination based on a DNA standard curve, normalized to the control group for every cell line and presented as a percent of stimulation.

#### Apoptosis assay

Activity of Caspase 3/7 was assessed using Caspase Glo 3/7 kit (Promega) according to the provided protocol. In brief, cells were seeded 8000/well on a 96-well plate, attached overnight and treated with CT (0.1% DMSO as control) or 10 µM CTX (CTX) for 6h. Absolute luminescence was normalized by CT-treated values.

#### GNRHR knockdown studies

To knockdown GNRHR in H295R cells, we delivered anti-GNRHR ON-TARGETplus SMARTpool (#L-005517-00-0005, Dharmacon) libraries using Lipofectamine RNAiMAX Transfection Reagent (Fisher Scientific). As a negative and positive control ON-TARGETplus Non-targeting Pool (#D-001810-10-05) and AllStars Hs Cell Death Control siRNA (#Sbib4381048, Qiagen) were used, respectively. Reverse transfection according to manufacturer’s protocol, in triplicates with a final 50 pmol siRNA concentration was performed and after overnight incubation medium was changed into full culture medium. After 72 h, each of the cell replicates were detached and seeded either onto 96-well plates for proliferation (*n* = 6/replicate) and caspase 3/7 activity studies (*n* = 3/replicate) or harvested for Western blot analysis (*n* = 1/replicate).

#### Western blot

Cells were rinsed with cold PBS and scraped on ice with RIPA buffer (Fisher Scientific) with addition of protease and phosphatase inhibitors (cOmplete ULTRA Tablets and PhosSTOP Phosphatase Inhibitor Cocktail Tablets, Roche). Equal amounts of total protein (20 μg) were separated on 10% polyacrylamide gels for 1.5 h at 100 V in 4°C. Proteins were transferred onto PVDF membranes using a semi-dry transfer for 30 min at 20 V. Membranes were blocked in 5% non-fat milk and incubated overnight with anti-GNRHR primary antibody (1:500; GNRHR03, MA5-11538, Invitrogen) at 4°C. Next day, membranes were washed in TBST and anti-mouse HRP-linked secondary antibody (Abcam) was applied for 30 min at RT. Amersham Biosciences ECL detection system (GE Healthcare, Little Chalfont, UK) was used for signal visualization. Images were taken using ChemiDoc MP imager (Bio-Rad). After visualization membranes were stripped using ReBlot Plus Strong Antibody Stripping Solution (Sigma-Aldrich), blocked in 5% non-fat dry milk and incubated overnight (4°C with gentle agitation) with primary anti-actin beta (ACTB) antibody (1:1000; A2228, Sigma-Aldrich). Intensity of the signals was calculated using ImageJ (NIH) software based on the measurements of three membranes.

### In vivo

In this study, we used inhα/Tag (transgenic mice expressing SV40 Tag oncogene under the inhibin α promoter) (Kananen* et al.* 1995, 1996) males and females (*n* = 6–8/gender/group). To induce adrenocortical tumors, animals were prepubertally (in between 21 and 24 days of their lives) gonadectomized under Isoflurane anesthesia (Isoflo, Orion, Finland). Temgesic (buprenorphine, 0.1 mg/kg/8 h) (Schering-Plough, Brussels, Belgium) and Comforion (ketoprofen, 5 mg/kg/24 h) were administered subcutaneously as a post-operative analgesia. Mice were raised in a pathogen-free media surrounded with controlled light (12 h light: 12 h darkness) and temperature (21 ± 1°C). Mice were housed two to five per cage, fed with mouse chow SDS RM-3 (Whitham, Essex, UK) and tap water *ad libitum*. Ethics Committees for animal experimentation of the Turku University and the State Provincial Office of Southern Finland approved all the animal experiments (ESAVI/3324/04.10.07/2014).

#### Preparation of drugs and treatments

Cetrorelix acetate (CTX, Sigma-Aldrich) was dissolved in sterile water and 0.75 mg/mL stock solution, kept in +4°C and used out within 6 days after reconstitution. Pregnyl 5000 IU (Merck) was dissolved in 99 mL of Natriumchlorid B. Braun 9 mg/mL (#630966, Braun, Melsungen, Germany), aliquoted into 1 mL syringes and frozen in −20°C. Single dose of cetrorelix acetate was 3 mg/kg/48 h, whereas Pregnyl 5 IU/30 g/week. At the age of 6.5 months, GDX inhα/Tag mice were randomly divided into experimental groups (*n* = 6–8/group/gender) and injected intraperitoneally for 21 days with weight-dependent amount of substance (1): saline as control (CT; Natrium chloride B. Braun 9 mg/mL (Braun)) (2), CTX (3), CTX together with Pregnyl (CTX + hCG) or (4) Pregnyl only (hCG). On day 23, mice were killed by exsanguination under isoflurane anesthesia. Blood was collected into a tube consisting 0.5 M sterile EDTA solution, and plasma was separated by centrifugation at 1800 ***g*** for 10 min in 4°C, and stored in −80°C for hormone measurements. After taking the weights, tissues were snap-frozen in liquid nitrogen or fixed with 4% paraformaldehyde.

#### RNA isolation and gene expression analysis

Total RNA from mouse adrenal tumors (*n* = 5/group/sex), 3 passages of cell lines (Cα1, Y-1, H295R), 11 adrenocortical carcinomas, 2 healthy adrenal glands or Cα1 and Y-1 cells treated for 24 h with control (CT; 0.1% DMSO as control) and CTX was extracted using TRIsure reagent (Bioline Reagents Ltd., London, UK) according to manufacturer’s protocol. RNA was quantified with Nanodrop (Fisher Scientific) and qualified by gel electrophoresis. Nine hundred nanograms of total RNA were treated with Amplification Grade DNase I (#AMPD1-1KT, Sigma-Aldrich) and transcribed (1 h in 48°C) using SensiFAST cDNA Synthesis Kit (#BIO-65053, Bioline Reagents Ltd.). qPCR reaction was consisting Fast SYBR Green Master Mix (Fisher Scientific), 7.5 ng of DNA template and primers from 

### In silico

To study global gene expression changes affected by CTX treatment, we compared adrenals of GDX inhα/Tag mice after 21-day treatment with CTX or CT (*n* = 4/group).

Total RNA was extracted using RNeasy Mini Kit (Qiagen) according to manufacturer’s protocol and qualified using Bioanalyzer nano kit (Agilent Technologies). cDNA was synthetized using the MessageAmp II aRNA Amplification Kit (Applied Biosystems) and ran on Agilent whole mouse genome oligo microarrays 4X44K (#GPL7202, Agilent Technologies, Santa Clara, CA, USA) accordingly to manufacturer’s protocol. Quantile normalization with Iimma R/Bioconductor package and a row-wise *t*-test were performed. Genes with fold-change higher than 2 and *P*-value lower than 0.05 were considered significant. Targets with *P*-value <0.1 and fold-change <1.2 for males and females were compared in Venn diagram (http://bioinfogp.cnb.csic.es/tools/venny/). List of treatment affected targets was uploaded to the PANTHER classification system (Mi* et al.* 2013) and statistical overrepresentation test was performed. Genes were classified using PANTHER GO-Slim Biological Processes and PANTHER pathways annotation data sets.

### Statistical analysis

To detect statistical differences between two or more experimental groups, we used Mann–Whitney *U* test or Kruskal–Wallis test with multiple comparison of mean range as *post hoc* analysis respectively. Numerical data were presented as mean ± s.e.m. Statistical analysis and graphs were prepared using Graph Pad Prism 6 (GraphPad Software), and *P* < 0.05 values were considered as significant.

## Results

### Expression of GNRHR, LHCGR and FSHR in adrenocortical tumors and cell lines

**Figure 1:**
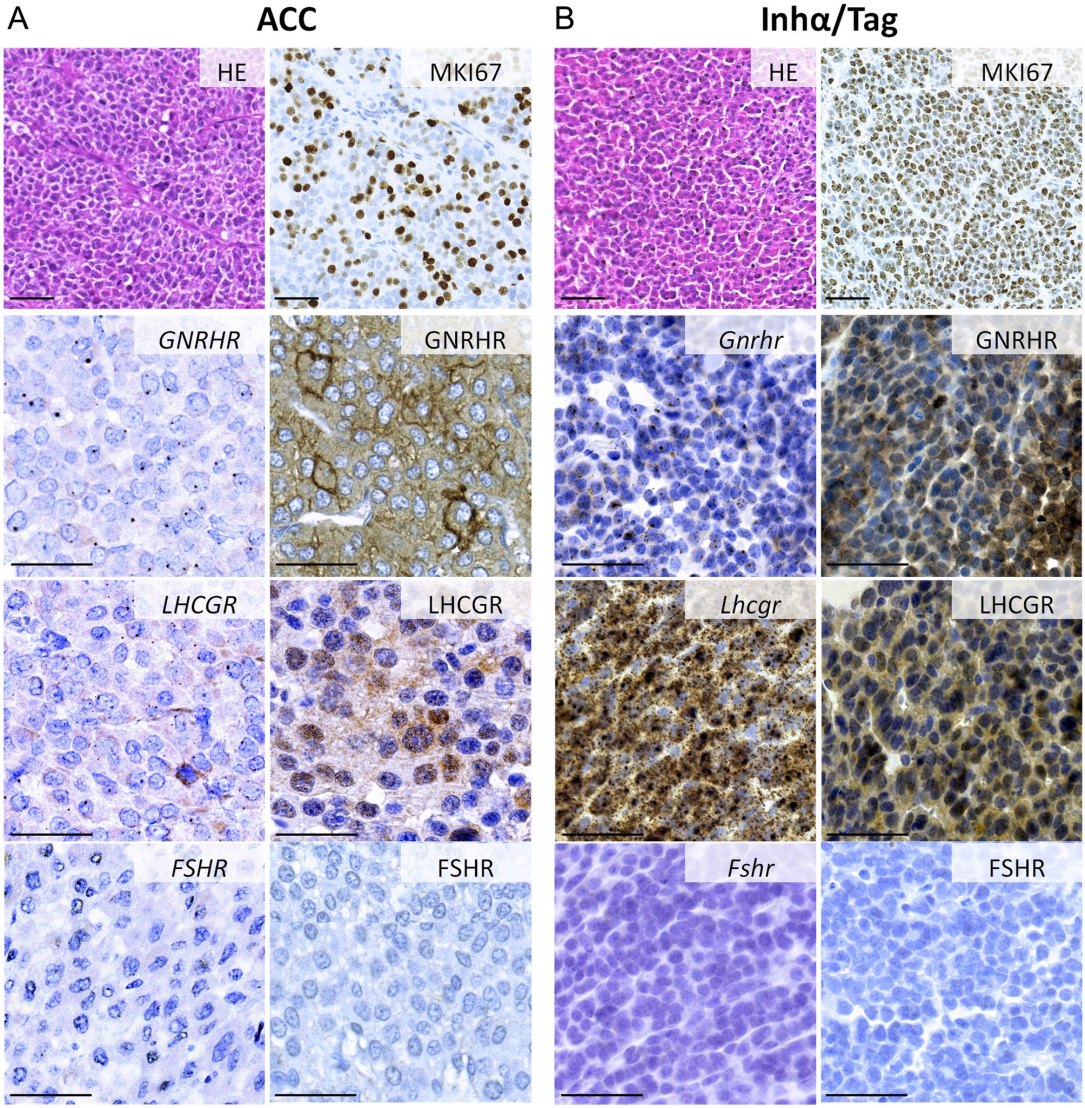
Localization of gonadotropin receptors in human and mouse adrenocortical tumors/cancer. Representative images of hematoxylin and eosin, immuno-localization of MKI67, *in situ* RNA/protein localization of *GNRHR*, *LHCGR* and *FSHR* in human adrenocortical carcinoma sections from Patient 11 (panel A) or adrenocortical tumor sections of inhα/Tag mouse (panel B). Bar = 50 µm.

**Figure 2:**
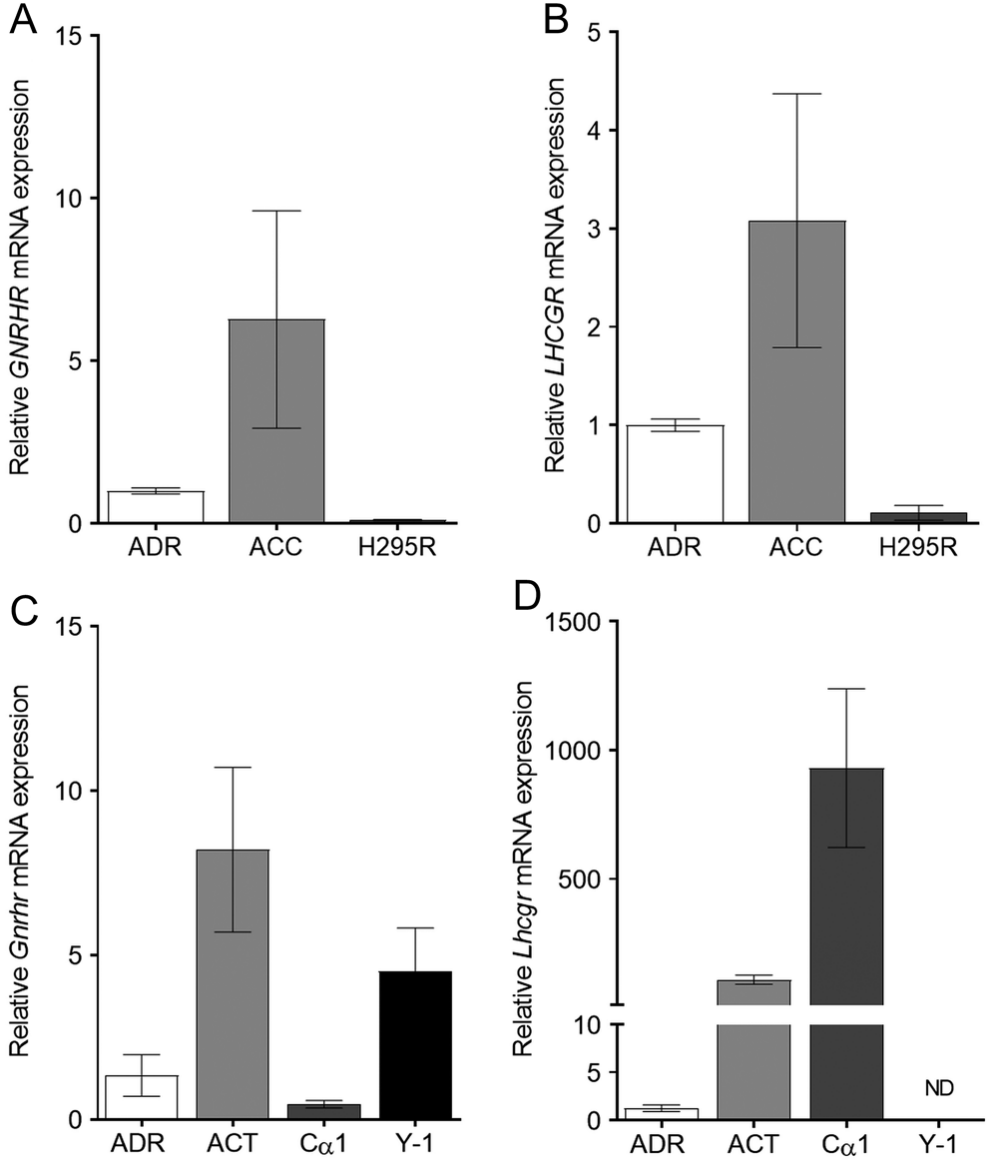
mRNA levels of *GNRHR/Gnrhr* and *LHCGR*/*Lhcgr* in normal and adrenal tumor/cancer tissues and cell lines. Expression of *GNRHR* (A) and *LHCGR* (B) in normal human adrenals (ADR) (*n* = 3), adrenocortical carcinomas (ACC) (*n* = 11) and H295R cell line (*n* = 3). Expression of *Gnrhr* (C) and *Lhcgr* (D) in normal mouse ADR (*n* = 5), ACT (*n* = 5) and cell lines Cα1 and Y-1. ACC, adrenocortical cancer, ACT, adrenocortical tumor; ADR, normal adrenal.

We determined the mRNA and protein expression of *GNRHR*, *LHCGR* and *FSHR* genes in samples from 13 patients, 6 males and 7 female patients, 30–72 years of age, presenting with diverse hormone excess manifestations (Table 1). Adrenocortical carcinoma samples were classified according to the European Network for the Study of Adrenal Tumors (ENSAT) scale from 2 to 4, and MKI67 proliferation index spanning between 2 and 50% (Table 1). In order to assess whether the adrenocortical carcinoma samples express *GNRHR*, *LHCGR* and *FSH* expression was studied using a commercial *in situ* hybridization RNAscope kit with single transcript resolution (Wang *et al.* 2012). We detected *GNRHR* in 54%, *LHCGR* in 77% and *FSHR* in 0% of the cancer samples (Table 1). The representative images of hematoxylin and eosin, immunohistochemical staining of MKI67, and localization of GNRHR/GNRHR, *LHCGR/*LHCGR and FSHR/FSHR transcripts and proteins in the human adrenocortical malignancies and in inhα/Tag adrenals are presented in the left (Fig. 1A) and right (Fig. 1B) panel, respectively. qPCR confirmed the expression of *GNRHR*/*Gnrhr* (Fig. 2A and C) and *LHCGR*/*Lhcgr* (Fig. 2B and D) in the normal human and mouse adrenals, adrenocortical tumors and H295R, Cα1, Y-1 cell lines (except *Lhcgr* in Y-1).

**Table 1:** Functional characterization of human adrenocortical carcinoma samples (*n* = 13) towards their *GNRHR*, *LHCGR*, *FSHR* mRNA and protein expression, proliferation and hormone secretion.

Patient number	Sex	Age	Stage (ENSAT)	Size (cm)	Hormone secretion	MKI67 (%)	*GNRHR/*GNRHR	*LHCGR/*LHCGR	*FSHR/*FSHR
1	Male	46	2	9.5	Aldosterone	20	**+**	**+**	**−**
2	Female	60	2	13	Androgen	20	**+**	**+**	**−**
3	Female	30	4	17	Androgen	40	**+**	**+**	**−**
4	Female	72	4	8	Cortisol	20	**−**	**−**	**−**
5	Female	57	4	12	Cortisol + androgens	20	**+**	**+**	**−**
6	Male	31	2	11	No clinical evidence	2	**−**	**+**	**−**
7	Female	40	2	21.5	No clinical evidence	10	**−**	**−**	**−**
8	Male	50	2	20	No clinical evidence	7	**+**	**+**	**−**
9	Female	46	3	18	No clinical evidence	40	**−**	**+**	**−**
10	Male	44	4	13	No clinical evidence	10	**−**	**−**	**−**
11	Female	44	4	4.5	Slight cortisol	50	**+**	**+**	**−**
12	Male	64	4	16	Slight cortisol	10	**−**	**+**	**−**
13	Male	59	4	5.5	Slight cortisol + androgen precursors	10	**+**	**+**	**−**

### CTX decreased cell viability and proliferation *in vitro* and tumor size *in vivo*

**Figure 3:**
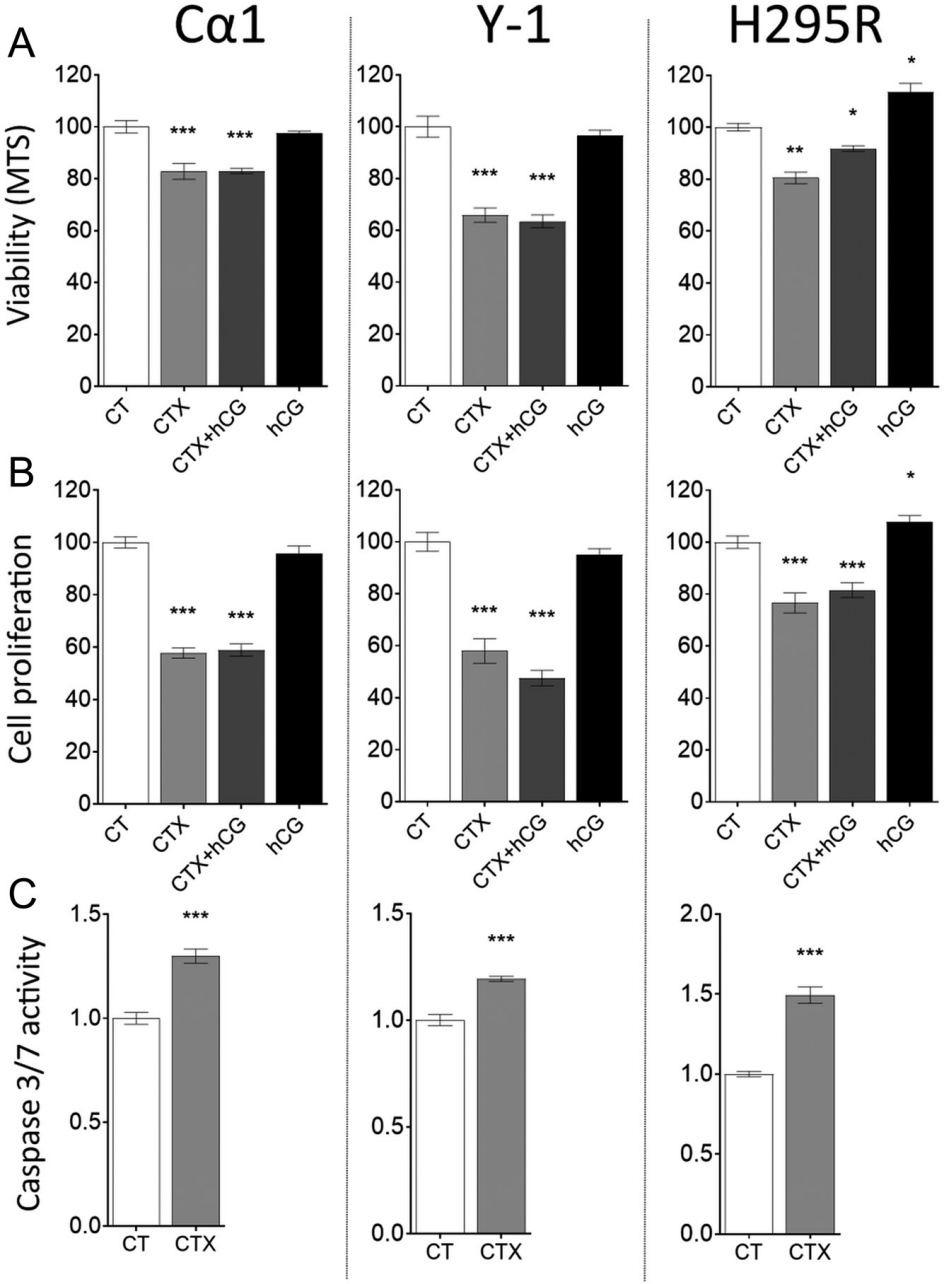
Cell viability, proliferation and caspase 3/7 activity *in vitro*. Viability (A) and proliferation (B) of Cα1, Y-1 and H295R cells after 48 h treatment with CT, CTX, CTX and hCG or hCG alone. Caspase 3/7 activity (C) in Cα1, Y-1 and H295R cells assessed after culture with CTX for 6 h (mean ± s.e.m.; *P ≤ 0.05. **P ≤ 0.01. ***P ≤ 0.001). CT, 0.1% DMSO as control; CTX, cetrorelix acetate; hCG, human chorionic gonadotropin.

**Figure 4:**
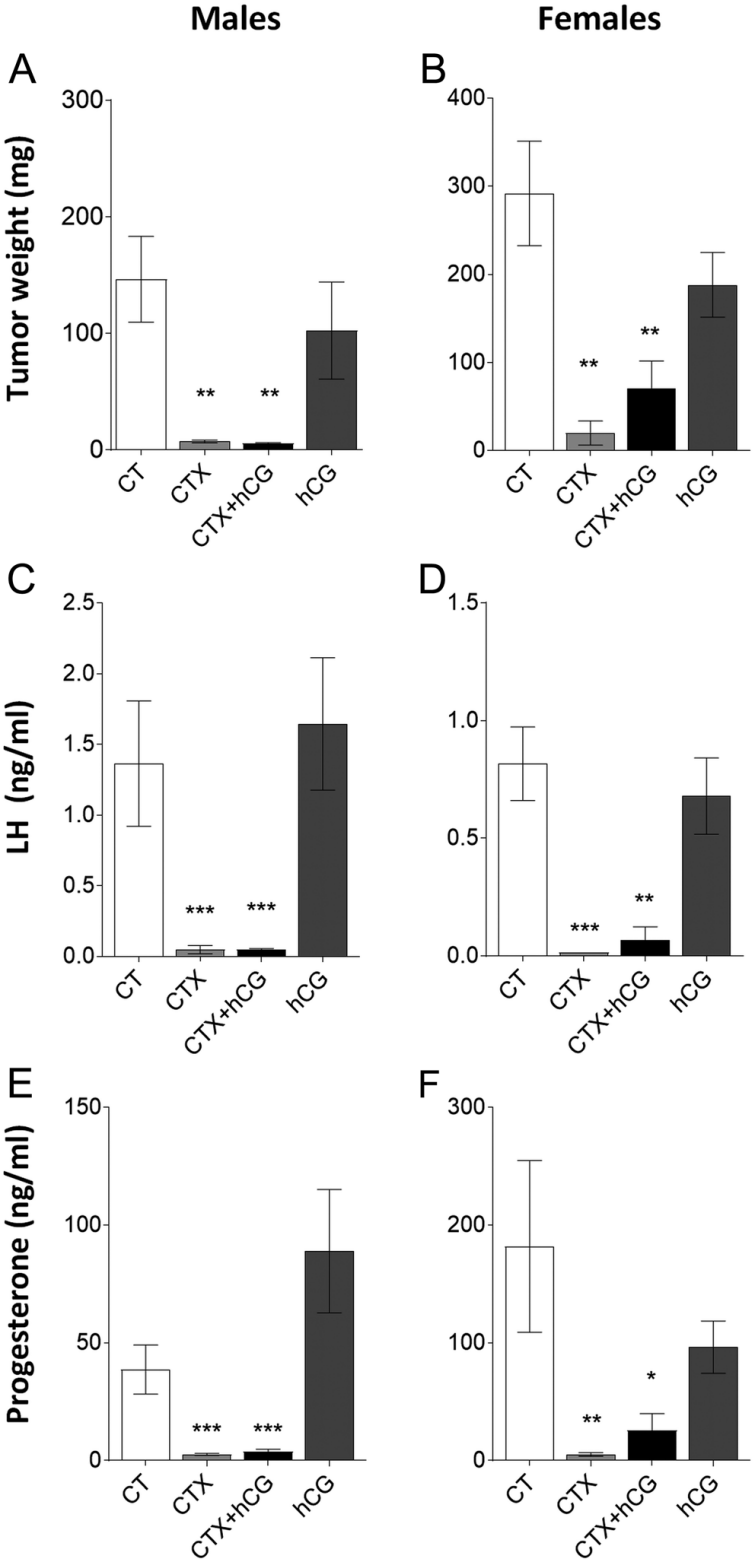
CTX treatment efficacy *in vivo*. Adrenal tumor weights (A and B) and plasma LH (C and D) and progesterone (E and F) concentrations of mice treated for 21 days with saline as control (CT), CTX, CTX and hCG, or hCG. (*n* = 6–8/group; mean ± s.e.m.; *P ≤ 0.05. **P ≤ 0.01. ***P ≤ 0.001). CT, saline; CTX, cetrorelix acetate; hCG, human chorionic gonadotropin.

To evaluate the potential tumor-regressing effect of CTX, we treated adrenocortical tumor cells *in vitro* and *in vivo*. To discriminate between direct and indirect CTX effects, we maintained LHCGR stimulation with hCG treatment. CTX alone or in combination with hCG (CTX + hCG) decreased the viability (Fig. 3A) and proliferation (Fig. 3B) of Cα1, Y-1 and H295R cells, whereas hCG alone significantly increased the viability and proliferation of H295R cells only (Fig. 3A and B). Moreover, CTX treatment increased the activity of caspase 3/7 in all treated cell lines (Fig. 3C). *In vivo* CTX and CTX + hCG treatments decreased adrenocortical tumor size of male (Fig. 4A) and female (Fig. 4B) mice as well as decreased LH (Fig. 4C and D) and progesterone (Fig. 4E and F) plasma concentrations when compared to CT. hCG treatment alone had no impact on the tumor size or plasma hormone concentrations (Fig. 4C, D, E and F).

### CTX acts through GNRHR expressed in tumor cells

**Figure 5:**
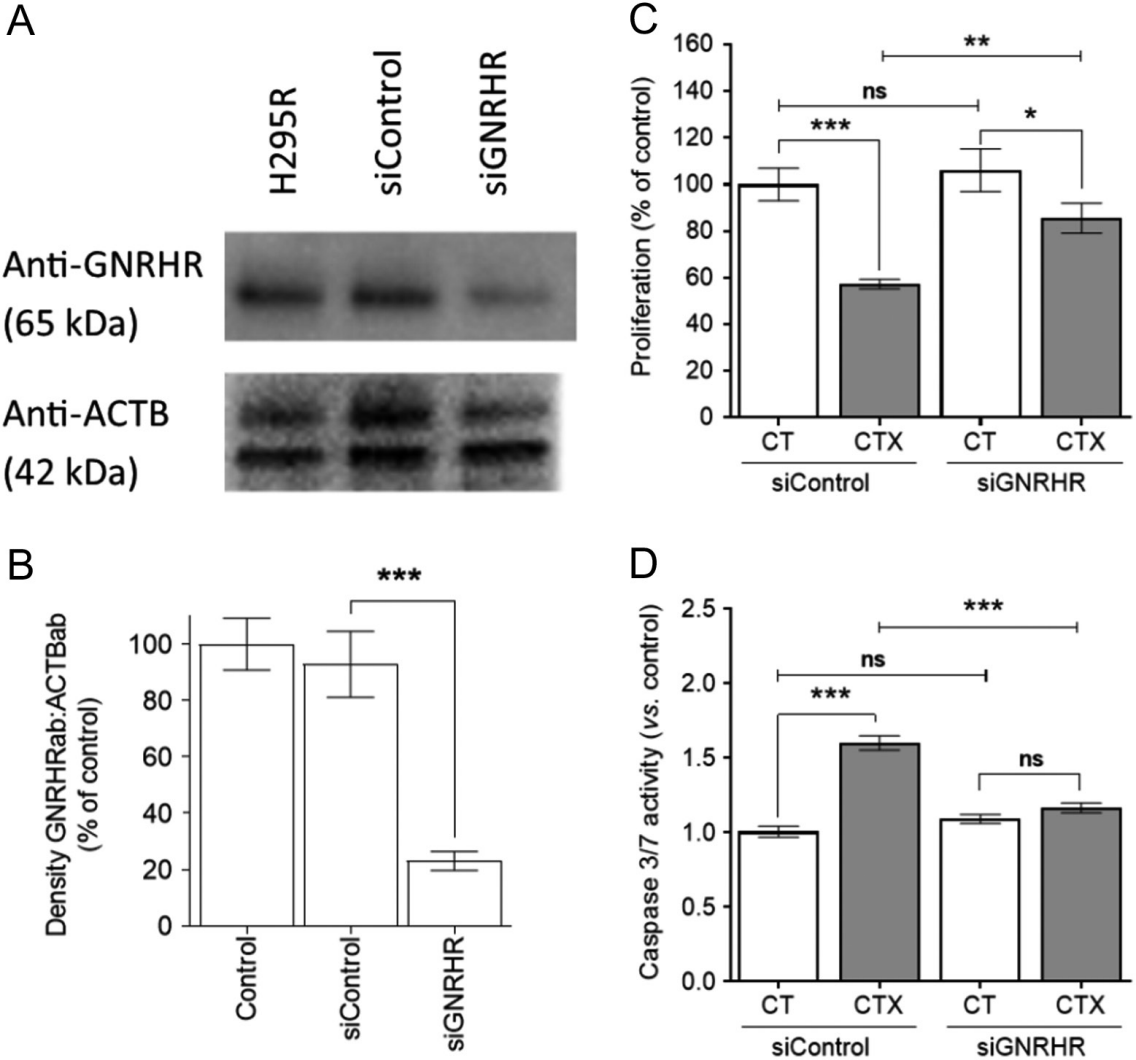
GNRHR knockdown in H295R adrenocortical carcinoma cells. Western blot analysis of GNRHR expression in WT (H295R), transfected with non-targeting (siControl) or GNRHR-targeting (siGNRHR) siRNA H295R adrenocortical carcinoma cells (A). Densitometric analysis of the GNRHR protein levels in H295R, siControl and siGNRHR cells (*n* = 3) (B). The effect of CTX treatment on proliferation (*n* = 12) (C) and caspase 3/7 activation (*n* = 9) (D) in siControl and siGNRHR cells (mean ± s.e.m.; *P ≤ 0.05. **P ≤ 0.01. ***P ≤ 0.001).

To confirm that the effect of CTX on adrenocortical carcinoma cells acts through GNRHR, we performed siRNA-mediated GNRHR knockdown in human H295R cells. Western blot analysis (Fig. 5A) revealed 78% knockdown of GNRHR protein levels (siGNRHR), as compared to cells transfected with non-targeting siRNA libraries (siControl) (Fig. 5B). Basal proliferation between siGNRHR and siControl cells was similar, and after 48-h CTX treatment, the proliferation of both was decreased (Fig. 5C). However, the inhibition of siGNRHR cell proliferation was significantly greater than that of siControl cells (Fig. 5C). Caspase 3/7 activity was similar in siGNRHR and siControl, but after CTX treatment significantly increased only in siControl cells (Fig. 5D).

### Global gene expression changes of adrenocortical tumors in inhα/Tag mice

**Figure 6:**
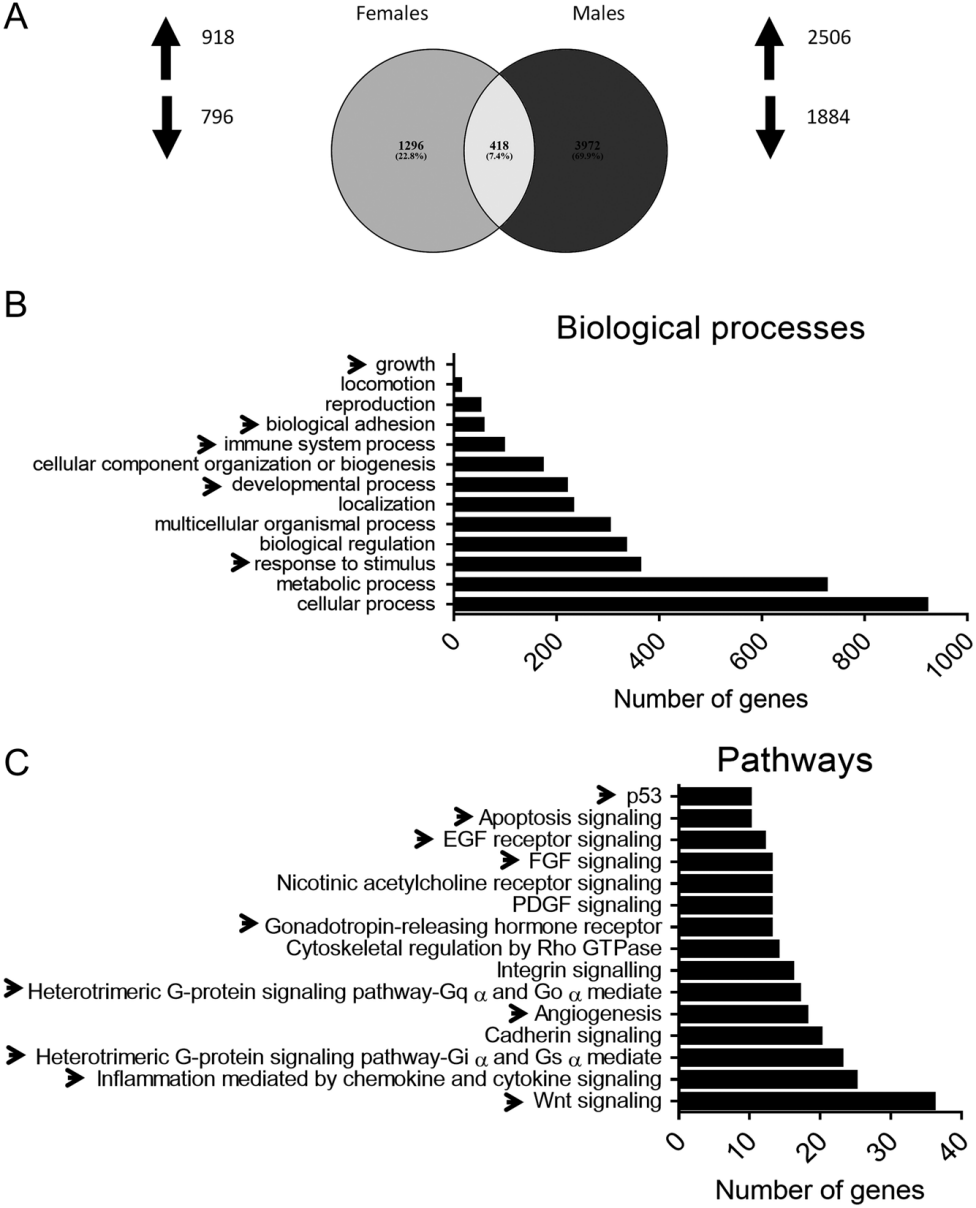
Gene ontology (GO) classification based on biological processes and pathways affected by CTX treatment *in vivo*. Venn diagram of upregulated and downregulated genes in the adrenal glands of male and female mice treated with CTX (cetrorelix acetate) or CT (saline as control) for 21 days (A). Significantly upregulated or downregulated genes were classified based on the biological processes (B) and pathways (C) they are involved in. The analysis shows the number of dysregulated genes in the adrenocortical tumors of inhα/Tag treated with CT vs CTX.

cDNA microarray analyses were run to identify the potential biological processes and pathways affected by CTX in ACTs of inhα/Tag mice. Due to high individual variation of tumor sizes, we considered fold change of ±1.2 and *P* value <0.1 significant. Complete cDNA microarray data can be found through ArrayExpress (accession number E-MTAB-5310). In females, CT vs CTX, 1714 genes were differently expressed, 918 upregulated and 796 downregulated. In males, CT vs CTX, 4390 genes were altered, 2506 up- and 1884 downregulated (Fig. 6A). As the individual variation of tumor size was lower in males than in females, we used the male dataset for further processing. Enrichment analysis with PANTHER classification system was used to cluster genes into biological processes and pathways. The most interesting processes (marked with arrowheads) were growth, biological adhesion, immune system, development and response to stimulus (Fig. 6B). Among dysregulated pathways, the most conspicuous (marked with arrowheads) were p53, apoptosis signaling, EGF receptor signaling, FGF signaling, gonadotropin-releasing hormone receptor, G-protein signaling pathways, angiogenesis, inflammation mediated by chemokine and cytokine signaling and Wnt signaling pathways.

### Gene expression signatures of CTX treatment in adrenocortical tumors of inhα/Tag mice

**Figure 7:**
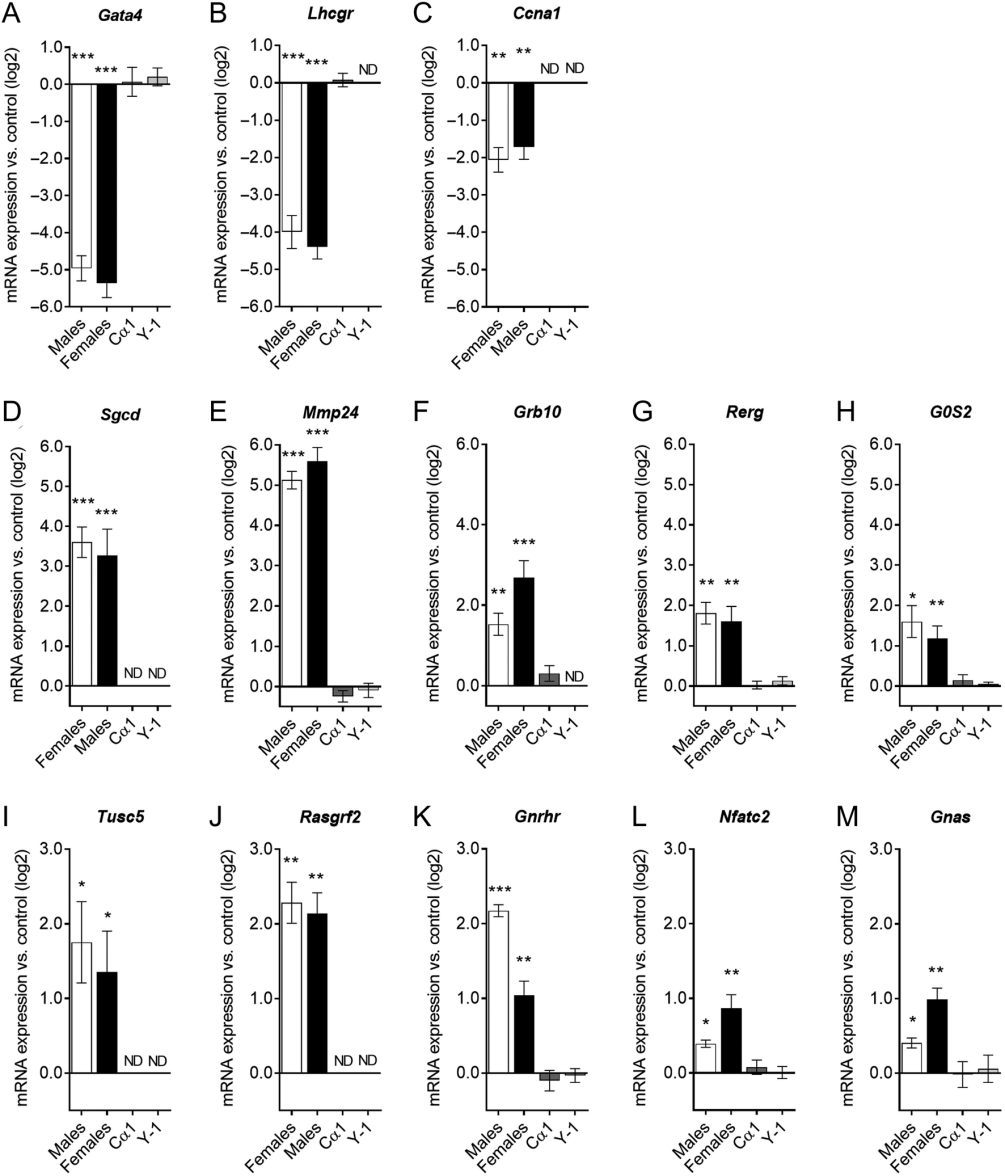
qPCR validation of selected genes from microarray study in inhα/Tag adrenal tumors and cell lines (Cα1 and Y-1) treated with CTX vs CT. Gene expression of *Gata4* (A), *Lhcgr* (B), *Ccna1* (C), *Sgcd* (D), *Mmp24* (E), *Grb10* (F), *Rerg* (G), *G0s2* (H), *Tusc5* (I), *Rasgrf1* (J), *Gnrhr* (K), *Nfatc2* (L), *Gnas* (M) in inhα/Tag adrenal tumors and Cα1 and Y-1 cell lines treated with CTX (cetrorelix acetate) vs CT (0.1% DMSO as control). (*n* = 3–5/group; mean ± s.e.m.; *P ≤ 0.05. **P ≤ 0.01. ***P ≤ 0.001).

Previously, we reported an array of genes up- or downregulated in adrenocortical tumors of inhα/Tag mice, when compared to WT littermates (Doroszko *et al.* 2017*a*). Interestingly, a subset of the genes such as transcription factor GATA4 (*Gata4*), *Lhcgr*, sarcoglycan delta (*Sgcd*), matrix metallopeptidase 24 (*Mmp24*), growth factor receptor-bound protein 10 (*Grb10*), RAS-like estrogen-regulated growth inhibitor (*Rerg*), gonadotropin-releasing hormone receptor (*Gnrhr*), G-protein-coupled receptor nuclear factor of activated T cells, calcineurin-dependent 2 (*Nfatc2*) and guanine nucleotide-binding protein alpha-stimulating complex locus (*Gnas*) also appeared in this microarray analysis. qPCR validation of the microarray analysis in murine adrenocortical tumors treated with CTX vs CT showed downregulation of *Gata4* (Fig. 7A), *Lhcgr* (Fig. 7B), Cyclin A1 (*Ccna1*) (Fig. 7C) and upregulation of extracellular matrix compounds such as *Sgcd* (Fig. 7D), *Mmp24* (Fig. 7E); genes related to cell growth suppression, *Grb10* (Fig. 7F), *Rerg* (Fig. 7G); G0/G1 Switch 2 (*G0s2*) (Fig. 7H); Tumor Suppressor Candidate 5 (*Tusc5*) (Fig. 7I) or GPCR-mediated Ras protein-specific guanine nucleotide-releasing factor 1 (*Rasgrf2*) (Fig. 7J). In addition, we found upregulation of *Gnrhr* (Fig. 7K) and the downstream mediators of G-protein-coupled receptor *Nfatc2* (Fig. 7L) and *Gnas* (Fig. 7M). Interestingly, none of these genes were altered in CTX-treated cells *in vitro* (Fig. 7A, B, C, D, E, F, G, H, I, J, K, L and M).

## Discussion

In recent years, several studies have detected the expression and function of the two reproductive hormone receptors, GNRHR and LHCGR, in non-gonadal tissues and malignancies (Alevizaki* et al.* 2006, Bernichtein* et al.* 2008, Lacroix* et al.* 2010, Banerjee & Fazleabas 2011, Limonta* et al.* 2012). In adrenal glands, gonadotropins, especially LH, may exert hormonal and hyperplastic effect (Carlson 2007, Plöckinger* et al.* 2017) as well as induce various adrenocortical pathologies. GNRHR expression has also been found in adrenocortical adenomas producing aldosterone (Albiger* et al.* 2011). Previously, *GNRHR* localization was shown in normal adrenal glands (medulla and cortex), adrenocortical adenomas and in the SW13 adrenocortical cell line, but never in adrenocortical carcinomas (Ziegler* et al.* 2009). LHCGR expression has been demonstrated in normal human adrenals (Pabon* et al.* 1996) and in various adrenocortical malignancies (Carlson 2007). FSHR has been found only by immunohistochemical localization in the intra- and peri-tumoral vessels of adrenal tumors (Pawlikowski* et al.* 2012). Reliable localization of gonadotropin receptors is challenging due to a lack of good-quality commercially available antibodies. Therefore, in our studies, we analyzed their expression levels by qPCR, mRNA localization in the tissue sections by a commercial *in situ* hybridization RNAscope kit and protein localization using in-house antibodies. We found that human adrenocortical carcinomas and mouse adrenocortical tumors expressed *GNRHR* and *LHCGR* mRNA and protein at variable levels, probably due to the well-known high heterogeneity of the tumors (Allolio & Fassnacht 2006). However, we were unable to detect *FSHR* transcripts or protein, in contrast to an earlier report (Pawlikowski* et al.* 2012), which used only immunohistochemistry. The inconsistency between RNA levels and protein detection using poorly characterized antibodies against FSHR is well recognized, necessitating RNA and protein measurements in tandem to ensure specificity and sensitivity of the FSHR detection (Stelmaszewska* et al.* 2016).

The present *in vivo* and *in vitro* data unequivocally showed that CTX could act directly on tumors causing their regression. CTX treatment decreased cell viability and proliferation as well as increased apoptosis in mouse and human adrenocortical tumors and cancer cell lines. GNRHR knockdown to reduce CTX action ultimately confirmed the on-target CTX effect. Similar direct GnRH antagonist effects on proliferation and cell death have been reported in prostate (Sakai* et al.* 2015), breast, uterine, lung (Ghanghoria* et al.* 2016) and adrenocortical (Ziegler* et al.* 2009) cell lines. Previous CTX treatment of adrenocortical tumors in inhα/Tag mice (Vuorenoja* et al.* 2009) showed tumor regression, which was attributed to systemic effect through inhibition of LH release. In contrast, we found now that hCG treatment did not affect Cα1 cell proliferation or tumor growth *in vivo*, which supports our recent report that adrenocortical tumor progression in inhα/Tag mice may be gonadotropin independent (Doroszko* et al.* 2017*b*). Additionally, the systemic action of CTX treatment *in vivo* was manifested by reduced plasma concentrations of LH and progesterone levels. Combining hCG with CTX treatment did not affect the outcome, implying that the CTX effect was independent of gonadotropin suppression but rather a direct effect on the adrenal tumors. Previously, H295R cells have been shown to express LHCGR and respond to hCG stimulation by producing DHEAS (Rao* et al.* 2004). In the current study, we confirmed the *LHCGR* expression in H295R adrenocortical carcinoma cells and showed that hCG stimulation resulted in mitotic effect. These data suggest a scenario of combined CTX action by direct blockage of adrenocortical tumor progression and indirect suppression of LH and steroid hormone concentrations that could be used for the benefit of the patient.

Gonadectomized domestic ferrets are another model that develops malignant adrenocortical tumors with gonadal-like phenotype (Li* et al.* 1998). Similar to inhα/Tag mice, tumor onset in ferrets was attributed to LH action. Therefore, therapies with GnRH agonist (Wagner* et al.* 2001, 2005) or GnRH adjuvant (Miller* et al.* 2013) to block circulating gonadotropins and subsequent hormone excess have been effective. It would be interesting to validate the LH dependency of the tumor progression and the expression of GNRHR in the ferret adrenal tumor tissues, as they could serve as another model for adrenocortical carcinomas.

Although the anti-angiogenic, anti-metastatic and pro-apoptotic effects of GnRH antagonists are well documented (Ghanghoria* et al.* 2016), the molecular mechanisms of their action remains poorly understood. A study on prostate cancer cell lines, with a different GnRH antagonist, degarelix, showed rather discrete mRNA changes after *in vitro* treatment (Sakai* et al.* 2015). It was suggested that the initial effects of GnRH antagonist could be non-genomic, directly triggering apoptosis in the effector cells (Sakai* et al.* 2015). To investigate the molecular changes in tumor cells induced by CTX, we carried out microarray analyses supported by qPCR validation. Arrays of genes were found upregulated, such as (*Mmp24*, *Rerg*, *Grb10*, *Gnrhr*, *Gnas*, *Nfatc2* and *Sgcd*) and downregulated, such as (*Gata4* and *Lhcgr*), in agreement with a previous study where tumorous and WT adrenals were compared (Doroszko* et al.* 2017*a*). Moreover, we have identified downregulation of *Ccna1*, an important contributor to G1/S cell cycle transition in somatic cells (Ji* et al.* 2005) and upregulation of genes related to cell growth suppression, such as *G0s2* (Welch* et al.* 2009) and *Tusc5* (Konishi* et al.* 2003), *Rasgrf2* (Qiu* et al.* 2003) in the CTX vs CT-treated adrenals. Interestingly, none of the investigated genes was affected in the Cα1 and Y-1 cell lines treated with CTX. This suggests that the regulation of gene expression does not proceed in the same fashion when tumor cells are grown *in vitro*. Downregulation of the tumor biomarkers *Gata4* and *Lhcgr* could be a sign of regressing tumor cells. On the other hand, the upregulated *Grb10*, *Rerg*, *Gnas* and *Nfatc2*, recently localized in abundance in the healthy adrenal gland (Doroszko* et al.* 2017*a*), could be a sign of physiological and regenerative response to replenish healthy adrenal. Therefore, our findings support the idea that the GnRH antagonist functions as a safe antitumor treatment that selectively and directly kills adrenocortical tumor cells.

Taken together, our data suggest that the GnRH antagonist cetrorelix acetate acts directly on adrenocortical tumor cells causing their apoptosis. The compound could improve the therapy of both hormone-secreting and non-secreting adrenocortical carcinomas both through systemic and direct action.

## Supplementary Material

Supporting Table 1

## Declaration of interest

The authors declare that there is no conflict of interest that could be perceived as prejudicing the impartiality of the research reported.

## Funding

The authors would like to thank Erica Nyman and Marja-Riitta Kajaala from the Turku Center for Disease Modeling (TCDM) for technical assistance. This work was financially supported by Turku Doctoral Programme of Molecular Medicine (M D), Finnish Cultural Foundation (M D), Academy of Finland (N A R, J T), Sigrid Juselius Foundation (J T), ERVA grant from Turku University Hospital (J T) and Polish National Science Center grant 2015/17/B/N25/00636 (N A R).

## Author contribution statement

M D, M C, N A R designed the study concept; M D, M C, J S, T S, S A performed the experiments; all the authors (M D, M C, J S, T S, S A, U P, M Q, S W, I H, J T, N A R) analyzed and interpreted the results; M D, M C, I H, J T and N A R drafted the manuscript and all the authors have approved the final manuscript.
