# Supplementary material for: GnRH antagonist treatment of malignant adrenocortical tumors
Source: Endocr Relat Cancer. 2018 Aug 21;26(1):103–17. doi: 10.1530/ERC-17-0399 (PMC6215908; doi:10.1530/ERC-17-0399)
Supplement: Supporting Table 1 [file erc-26-103-t001.pdf]

Table S1. List of primers used in the experiment

Gene symbol		5′ --sequence-- 3′	bp	Name (synonyms)
Ppia*	F	CATCCTAAAGCATACAGGTCCTG	23	peptidylprolyl isomerase A (Cyclophilin A)
	R	TCCATGGCTTCCACAATGTT	20	
Gusb*	F	TGCCTGTCCCTTCTAGCTTC	20	glucuronidase, beta
	R	AATGGGCACTGTTGATCCTC	20	
Hprt1*	F	TGACACTGGTAAAACAATGCAA	20	hypoxanthine guanine phosphoribosyl transferase
	R	CAAAGTCTGGCCTGTATCCAA	20	
Ppib*	F	AGCCTTAGCTACAGGAGAGAAAGGA	25	peptidylprolyl isomerase B
	R	ATGCTCTTTCCTCCTGTGCCA	21	
Hmbs*	F	AAATCATTGCTATGTCCACCACG	23	hydroxymethylbilane synthase
	R	GCCAGGAGGTAGTATGGTAGGCA	23	
Gnrhr	F	CAAGACCCACGCAAACTACA	20	gonadotropin releasing hormone receptor
	R	TTTCTGGATCAAACCAGTACCA	22	
Lhcgr	F	CAATGGGACGACGCTAATCT	20	luteinizing hormone/choriogonadotropin receptor
	R	CTGGAGGGCAGAGTTTTCAG	20	
Gata4	F	AAGACGCCAGCAGGTCCTG	19	GATA binding protein 4
	R	AGTACTGAATGTCTGGGACATGGAG	25	
Ccna1	F	GGAAGCTGACCCATTCTTGA	20	Cyclin A1
	R	ATAGCCCGTAAATGCAGCAA	20	
Nfatc2	F	CACAGATACGGTGACCCCTG	20	nuclear factor of activated T cells, cytoplasmic, calcineurin dependent 2
	R	GTGCGATCGGTTCTTCTTCG	20	
Mmp24	F	CTGGGCAGAACTGGTTAAA	19	matrix metallopeptidase 24
	R	ATCCCGTAAAACTGCTGCAT	20	
Sgcd	F	TGTGGAAGCATATGGCAAAA	20	sarcoglycan, delta (dystrophin-associated glycoprotein)
	R	GACTGTGCCTTCAGCTCCTAA	21	
Gnas	F	TTGACTGTGCCCAGTACTTCC	21	GNAS (guanine nucleotide binding protein, alpha stimulating) complex locus
	R	ACATCGAACATGTGGAAGTTGA	22	
Grb10	F	TTGCCTTCGGTTTTTCTCC	19	growth factor receptor bound protein 10
	R	GTTGACTGCCCCACTTTGTC	20	
Rerg	F	AGTCACCAGATACCCTGGATTG	22	RAS-like, estrogen-regulated, growth-inhibitor
	R	TTCAGATTCCAGATGTGAGCA	21	
Tusc5	F	GGGCATCGTTATCATCATTG	20	Tumor Suppressor Candidate 5

	R	TGTGTGGGGACATGTAGGTT	20	
Rasgrf2	F	ACCGAAATCGAAAGGCTTAAA	21	Ras Protein Specific Guanine Nucleotide Releasing Factor 2
	R	TCCTACACAGCCACCCTCTC	20	
G0s2	F	CCCAGAGCTCAGATGGAAAG	20	G0/G1 Switch 2
	R	TGCACACCGTCTCAACTAGG	20	
Human primers				
GUS*	F	GCCATCGTGTGGGTGAATG	19	glucuronidase, beta
	R	GGATACTTGGAGGTGTCAGTCAGG	24	
PPIA*	F	GCCAAGACTGAGTGGTTGGATG	22	peptidylprolyl isomerase A (Cyclophilin A)
	R	GAGTTGTCCACAGTCAGCAATGG	23	
RN18S*	F	GAGGATGAGGTGGAACGTGTG	21	18S ribosomal RNA
	R	GTTGTCCAGACCATTGGCTAGG	22	
LHCGR	F	CTGAGTGGCTGGGACTATGA	20	luteinizing hormone/choriogonadotropin receptor
	R	CCAAATCAGGACCCTAAGGA	20	
GNRHR	F	GACCTTGTCTGGAAAGATCCGA	22	gonadotropin releasing hormone receptor
	R	GTCTCCAACAGGTTGGCTAAGG	22	
GNRH	F	GCCAATTCAAAAACTCCTAGCTG	23	Gonadotropin releasing hormone
	R	GACCAACCTCTTTGACTATCTCTTGG	26	

* were tested as a reference genes
